# Impact of an Altered Wnt1/β-Catenin Expression on Clinicopathology and Prognosis in Clear Cell Renal Cell Carcinoma

**DOI:** 10.3390/ijms140610944

**Published:** 2013-05-24

**Authors:** Stephan Kruck, Christian Eyrich, Marcus Scharpf, Karl-Dietrich Sievert, Falco Fend, Arnulf Stenzl, Jens Bedke

**Affiliations:** 1 Department of Urology, Eberhard Karls University Tuebingen, Hoppe-Seyler Strasse 3, Tuebingen 72076, Germany; E-Mails: stephan.kruck@med.uni-tuebingen.de (S.K.); ch.eyrich@gmx.de (C.E.); karl.sievert@med.uni-tuebingen.de (K.-D.S.); arnulf.stenzl@med.uni-tuebingen.de (A.S.); 2 Institute of Pathology, Eberhard Karls University, Tuebingen 72076, Germany; E-Mails: marcus.scharpf@med.uni-tuebingen.de (M.S.); falko.fend@med.uni-tuebingen.de (F.F.)

**Keywords:** renal cell cancer, Wnt1, β-catenin, carcinogenesis, prognosis, targeted therapy

## Abstract

In renal cell carcinoma (RCC), single members of the Wnt/β-catenin signaling cascade were recently identified to contribute to cancer progression. However, the role of Wnt1, one of the key ligands in β-catenin regulation, is currently unknown in RCC. Therefore, alterations of the Wnt1/β-catenin axis in clear cell RCC (ccRCC) were examined with regard to clinicopathology, overall survival (OS) and cancer specific survival (CSS). Corresponding ccRCCs and benign renal tissue were analyzed in 278 patients for Wnt1 and β-catenin expression by immunohistochemistry in tissue microarrays. Expression scores, including intensity and percentage of stained cells, were compared between normal kidney and ccRCCs. Data was categorized according to mean expression scores and correlated to tumor and patients’ characteristics. Survival was analyzed according to the Kaplan-Meier and log-rank test. Univariable and multivariable Cox proportional hazard regression models were used to explore the independent prognostic value of Wnt1 and β-catenin. In ccRCCs, high Wnt1 was associated with increased tumor diameter, stage and vascular invasion (*p* ≤ 0.02). High membranous β-catenin was associated with advanced stage, vascular invasion and tumor necrosis (*p* ≤ 0.01). Higher diameter, stage, node involvement, grade, vascular invasion and sarcomatoid differentiation (*p* ≤ 0.01) were found in patients with high cytoplasmic β-catenin. Patients with a high cytoplasmic β-catenin had a significantly reduced OS (hazard ratio (HR) 1.75) and CSS (HR 2.26), which was not independently associated with OS and CSS after adjustment in the multivariable model. Increased ccRCC aggressiveness was reflected by an altered Wnt1/β-catenin signaling. Cytoplasmic β-catenin was identified as the most promising candidate associated with unfavorable clinicopathology and impaired survival. Nevertheless, the shift of membranous β-catenin to the cytoplasm with a subsequently increased nuclear expression, as shown for other malignancies, could not be demonstrated to be present in ccRCC.

## 1. Introduction

Approximately 64,770 new diagnoses of kidney cancer are made each year, resulting in over 13,570 deaths reported in the United States, with a growing trend in 2012 [1]. Current therapeutic strategies are mainly based on the targeted inhibition of the vascular endothelial growth factor (VEGF) and mTOR pathway, rarely on the use of immunotherapy. These treatments improved patients’ median overall survival up to 26 months, but prognosis is still limited, as long-term responders are rare. Therefore, there is the definitive need for the improvement of therapeutic concepts, since progressive disease will develop, due to the inevitable development of a multi-drug resistance in metastatic renal cell carcinoma (mRCC) patients [2]. For future therapies of mRCC, the Wnt/β-catenin pathway, as a key regulator of cellular homeostasis in adult tissues and cell-to-cell interactions during embryogenesis, was proposed as a promising candidate [3–9]. Wnt/β-catenin signaling can be either categorized as canonical or non-canonical. Although after Wnt ligand binding in both pathways, signaling is transduced through frizzled receptor proteins along with co-receptors, only the canonical pathway activates the key regulator protein β-catenin by preventing its phosphorylation-dependent degradation [10]. As a consequence, increased cytoplasmic β-catenin levels result in reduced cadherin-based cell adhesivity increased epithelial-mesenchymal transition (EMT) and progressive cancer metastases [11]. For signal transduction, β-catenin binds to the N-terminus of the lymphocyte and T-cell enhancer factors (LEF/TCF) after translocation into the nucleus. Here, elevated transcription of LEF/TCF-responsive target genes, such as c-myc or cyclin D1, promote deregulated cell differentiation and apoptosis [10,12]. These mechanisms are particularly evident in colorectal and hepatocellular carcinomas, where frequent associations with gene mutations of the β-catenin regulator genes, adenomatous polyposis coli (APC) or AXIN, are found [13]. Furthermore, the Wnt/β-catenin pathway is closely linked to other key signaling pathways in RCC, including the PI3K/AKT [14] or the hypoxia-inducible factor [15] regulation. Although comprehensive investigations using genetic, epigenetic, transcriptomic or proteomic profiling elucidated β-catenin activation via canonical Wnt signaling as the central effector of cancer progression and metastasis, the significance of oncogenic signaling via Wnt ligands and β-catenin remains controversial in renal cancer [11]. In this context, the Wnt1 ligand, the first member of Wnt family proteins, does not only play a key role in the induction of kidney morphogenesis, but also seems to participate in renal tumorigenesis [16,17]. In particular, Wnt1 increases proliferation and inhibits apoptosis through the canonical pathway activation [18]. The overexpression of Wnt1 was reported in various human cancers and is also suggested to be involved in ccRCC progression, as observed in Wnt ligand screening in RCC cancer samples [8,19].

However, the tumorigenic and prognostic significance of Wnt1 has never been elucidated in ccRCC patients. Independent of a reported Wnt ligand stimulation, cytoplasmic accumulation of β-catenin seems to be restricted to ccRCC in contrast to papillary or chromophobe RCCs [4]. Nevertheless, reported ccRCC results concerning β-catenin protein expression also differ with regard to the localization in the membrane, the cytoplasm or the nucleus [3–8]. Until now, no study has been carried out to clarify the role of Wnt1 and β-catenin activation with special respect to clinicopathology, overall and cancer-specific survival. Therefore, this study addresses alterations in the Wnt1/β-catenin signal axis for potential future diagnostic and therapeutic approaches in ccRCC.

## 2. Results and Discussion

### 2.1. Results

#### 2.1.1. Patient Characteristics

The mean ± SD age of the 278 patients was 62.2 ± 12.5 years; 69.8% of patients were male. Patients were classified as ≤pT2 in 60.8% and ≥pT3 in 39.2%. Lymph node involvement was present in 13 patients (4.7%), and 39 (14.0%) patients had evidence of distant metastatic disease. Nuclear grading according to the Fuhrman classification was G1/2 in 234 (84.2%) patients and G3/4 in 44 (15.8%) patients (Table 1).

#### 2.1.2. Expression of Wnt1 and β-Catenin in Normal Kidney Tissue and ccRCC

**Figure 1:**
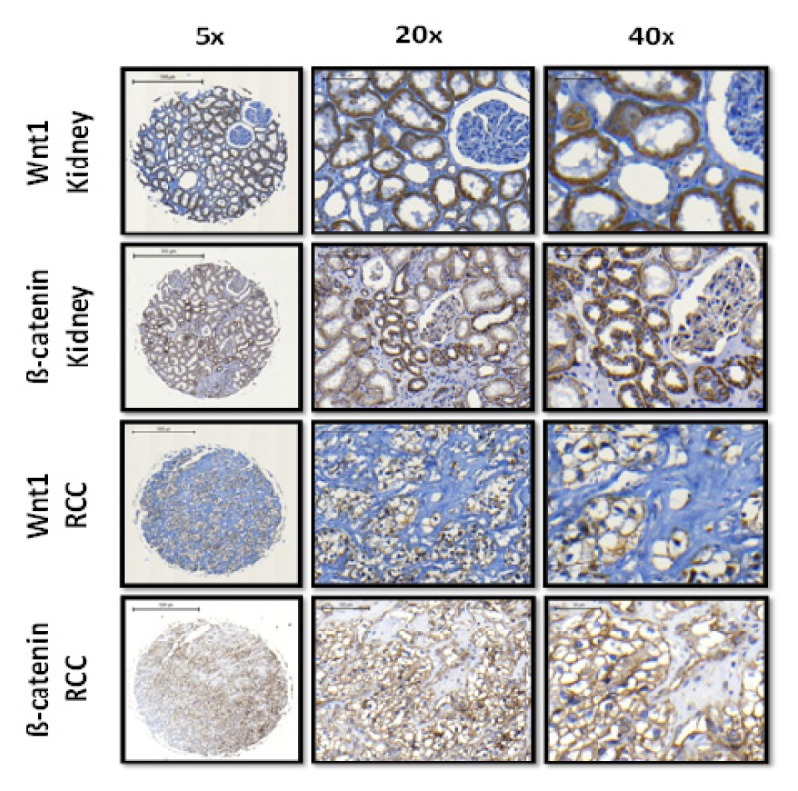
Representative immunohistochemical staining of Wnt1 and β-catenin in normal renal and ccRCC tissues.

Wnt1 expression was mainly present in the cytoplasm of proximal renal tubules. Higher expression of Wnt1 was found in normal kidney parenchyma (52.2 ± 22.6%) compared to ccRCC (31.0 ± 23.5%; *p* < 0.0001). β-catenin was mainly expressed in the membrane of proximal and distal tubules, while only minor reactivity was observed in the cytoplasm. Higher, albeit not significant, membranous β-catenin immunoreactivity was observed in benign renal samples (60.6 ± 12.8%) in comparison to the corresponding ccRCC tissue (55.8 ± 15.8%, *p* = 0.47). Furthermore, no significant cytoplasmic expression difference was found (kidney: 43.2 ± 14.3%; *vs*. ccRCC: 38.8 ± 13.5, *p* = 0.25). Interestingly, nuclear β-catenin immunoreactivity was only present in 18 (6.5%) of ccRCC samples in ≤5% of cancer cells; nuclear β-catenin expression was absent in benign kidney. Representative immunohistochemical staining of Wnt1 and β-catenin in normal renal and ccRCC tissues is shown in Figure 1.

#### 2.1.3. Correlation of Wnt1 and β-Catenin to Clinico-Pathologic Data in ccRCC

A higher Wnt1 expression (>mean tumor score of 40.0%) was associated with higher tumor diameter (5.8 cm ± 3.0 *vs.* 4.9 cm ± 2.6, *p* = 0.01), tumor stage (T3/4: 52.8% *vs.* T1/2: 30.9%, *p* = 0.004) and risk of vascular tumor infiltration (V1: 39.6% *vs.* V0: 24.9%, *p* = 0.02). A high membranous β-catenin (>mean tumor score of 69.0%) was related to a higher tumor stage (T3/4: 61.4% *vs.* T1/2: 36.0%, *p* = 0.03), a higher grading (G3/4: 24.3% *vs.* G1/2: 12.9%), a higher rate of vascular invasion (V1: 44.3% *vs.* V0: 26.3%, *p* = 0.01) and a higher number of tumor necrosis (52.9% *vs.* 38.7%, *p* = 0.04). A high cytoplasmic β-catenin (>mean tumor score of 37.5%) expression was positively correlated to a larger tumor diameter (6.4 cm ± 2.9 *vs.* 5.2 cm ± 2.7, *p* = 0.01), a higher tumor stage (T3/4: 71.0% *vs.* 36.0%, *p* = 0.003), the presence of lymph node involvement (12.9% *vs.* 3.6%, *p* = 0.04), a higher nuclear grade (G3/4: 48.4% *vs.* G1/2: 11.6%, *p* ≤ 0.001), the presence of vascular invasion (V1: 58.1% *vs.* V0: 27.6%, *p* = 0.002) and sarcomatoid differentiation (19.4% *vs.* 5.3%, *p* = 0.01). Analysis showed no significant findings for nuclear β-catenin expression (data not shown). Detailed results of Wnt1, membranous and cytoplasmic β-catenin expression are shown in Table 1.

#### 2.1.4. Correlation to OS and CSS of Wnt1 and β-Catenin

**Figure 2:**
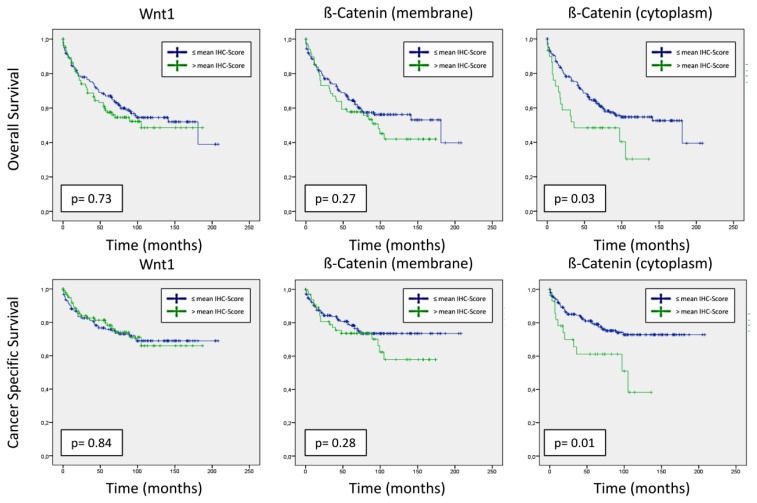
Kaplan-Meier survival analysis of Wnt1, membranous β-Catenin and cytoplasmic β-Catenin accumulative effects on overall survival and cancer-specific survival (log rank analysis). Patients were categorized by mean immunohistochemical (IHC) protein expression scores (blue line ≤ mean IHC score; green line > mean IHC score).

The median follow-up was 65 months (interquartile range: 20–100). There was no significant difference in median OS and CSS times [months] for patients with a low *vs.* a high Wnt1 protein expression [OS: 124.9 (109.7–140.2) *vs.* 109 (92.1–125.9), *p* = 0.73; CSS: 153.8 (139.7–167.9) *vs.* 138.9 (121.6–156.2), *p* = 0.84)) and low *vs.* a high membranous β-catenin expression (OS: 125.2 (110.3–140.0) *vs.* 97.1 (79.2–115.0), *p* = 0.27; CSS: 160.7 (147.8–173.7) *vs.* 121.4 (102.8–140.0), *p* = 0.28]. In contrast, a significantly longer OS and CSS was observed in patients with a low intratumoral cytoplasmic expression of β-catenin (OS: 124.9 (111.4–181.0) *vs.* 66.9 (45.7–88.1), *p* = 0.03; CSS: 160.9 (149.2–172.7) *vs.* 81.1 (58.8–103.5), *p* = 0.01)), as shown in Figure 2.

#### 2.1.5. Prognostic Value of Wnt1 and β-Catenin on Survival

In the Cox proportional hazard model (Table 2), the unfavorable clinicopathologic parameters (stage, lymph node status, evidence of distant metastasis, nuclear grade, tumor necrosis, sarcomatoid differentiation, vascular and sinus invasion, *p* ≤ 0.002), as well as higher cytoplasmic β-catenin (OS: *p* = 0.035; CSS: *p* = 0.012) were significantly associated with a higher risk of cancer-specific and non-cancer specific death. In addition, reduced OS was found in ccRCC patients with higher age above the median (≥64 years, *p* = 0.007) and perinephric invasion (*p* = 0.008).

If adjusted in the multivariable model (Table 3), an increased cytoplasmic β-catenin expression did not remain an independent predictor of overall or cancer-specific survival, while advanced stage (OS: HR: 2.63, *p* < 0.0001, CSS: HR: 4.30 *p* < 0.0001) and the evidence of distant metastasis (OS HR: 3.43, *p* < 0.0001, CSS: HR: 4.58, *p* < 0.0001) was. Higher age (HR: 1.88, *p* = 0.002) was only associated with a shorter OS.

### 2.2. Discussion

The members of the Wnt protein family are strongly implicated as regulators of mammary cell growth and differentiation [12]. The first discovered Wnt1 protein was identified as a virus-induced proto-oncogene in mouse mammary tumors in 1982 [20]. Later, multiple other Wnt family members have been detected in the human genome [21]. During the last decade, Wnt-related pathways have been substantially elucidated. Wnt proteins bind to a cell surface receptor complex, which consists of members of the frizzled receptor family and the low-density-lipoprotein receptor-related proteins, 5/6 [22]. After binding of the Wnt-ligands, β-catenin accumulates in the cytoplasm, due to the prevention of β-catenin degradation. Subsequently, nuclear shuttling of cytoplasmic β-catenin is required for activation of target genes, for instance, lymphocyte and T-cell enhancer factors (LEF/TCF), c-myc, VEGF or mTOR [23,24]. Co-culture experiments in esophageal cancer cells demonstrated that Wnt1, but not Wnt5A and Wnt7A, results in the activation of LEF/TCF-dependent transcription and β-catenin stabilization [25]. In addition, canonical Wnt/β-catenin signaling affects EMT by cadherin-mediated cell adhesion, which controls the expression of adhesion enzymes, mesenchymal genes and directly affects cell motility [26].

Many lines of evidence suggest that Wnt/β-catenin signaling is centrally involved in ccRCC tumorigenesis: First, the β-catenin coding gene is located on the short arm of chromosome 3, a region frequently affected by somatic alterations and associated with enhanced carcinogenesis, e.g., Von Hippel-Lindau (VHL)-mutations in renal cancers [27,28]. Although, mutations of β-catenin are relatively rare events in ccRCC, high cytoplasmic β-catenin protein levels were reported in ccRCC, which suggests that alternative mechanisms mediated via growth factors or hypoxia-induced (HIF) pathways might be responsible for an upregulated β-catenin [3,4,29]. Second, the loss of function of Wnt antagonist has been frequently reported to be associated with Wnt/β-catenin signal activation and RCC development [30–34]. In particular, the loss of secreted-Frizzled related proteins (sFRP) leads to increased cytoplasmic β-catenin levels in RCC experiments [33,34].

Furthermore, the downregulation of another Wnt-antagonist, the Wnt-inhibitory factor 1 (WIF-1), is frequently observed in RCC, while the restoration of WIF-1 suppresses tumor growth [35]. This phenomenon was also reported for a third group of Wnt pathway antagonists, the Dickkopf 1–3 proteins [36–38]. Whereas, the underlying mechanisms of Wnt/β-catenin regulation in cell culture experiments appear entirely conclusive, clinical studies in RCC showed widely differentiated results, especially regarding β-catenin protein expression and localization. The loss of membranous β-catenin correlated with advanced stages and nodal involvement, while no further correlation between β-catenin expression and cancer recurrence or survival was detected [3]. In another study, the membranous and cytoplasmic expression without nuclear β-catenin expression was confirmed for ccRCC. Accumulation of cytoplasmic β-catenin was only observed in 22.7% of 22 ccRCC patients and was completely absent in all papillary or chromophobe cancers [4]. In contrast, no difference in β-catenin mRNA levels was present in tumor samples compared with normal tissue, as observed in another series [9]. Later, in a larger study of 124 RCC patients, nuclear β-catenin expression was detected in 14% of tumors without any statistical significance. However, a decreased membranous β-catenin expression was associated with extensive local RCC growth and was identified as an independent predictor for a short recurrence-free survival [5]. In another study, β-catenin was predominantly detected in the cytoplasm of proximal and distal kidney tubules. Here, the majority of ccRCCs showed strong cytoplasmic or membranous expression. However, no nuclear staining was observed in malignant and normal tissues in this series [6].

In contrast, other investigators found positive nuclear β-catenin immunostaining in 44% of 152 RCC patients and associated nuclear immunoreactivity with lower Fuhrman grades [39]. Recently, a study using real-time polymerase chain reaction and immunohistochemistry in 60 RCC specimens confirmed higher cytoplasmic β-catenin protein levels in undifferentiated RCCs. In correspondence to Zang *et al*., again, no significant tumor-specific differences in mRNA levels of β-catenin were reported. Elevated cytoplasmic β-catenin expression was present in 91.7% of ccRCC patients and was associated with a shorter overall survival [7]. Very recently, Hsu and co-workers performed Wnt ligand screening in RCC tissue and cell lines and identified the most overexpressed Wnt ligand, 10A, as an oncogene responsible for disease progression via the activation of β-catenin signaling. The performed ligand screening identified Wnt1 as the second most expressed Wnt ligand type in RCC tissue samples, without reporting any further related clinical or survival data [8].

Taken together, there seems to be great heterogeneity in the reported data, especially concerning localization-dependent β-catenin protein expression in comparison to normal kidney and their function in RCC carcinogenesis.

While membranous and cytoplasmic β-catenin expression is frequently associated with unfavorable RCC biological and clinical behavior, nuclear localization is only inconsistently reported [3–7]. In accordance our data showed a close association between Wnt1, membranous and cytoplasmic β-catenin and unfavorable clinico-pathologic features, such as tumor diameter, stage, Furman grade, vascular invasion, sarcomatoid differentiation and node involvement, and also reflected by reduced OS (*p* = 0.03) and CSS (*p* = 0.01) in cancers with high β-catenin levels in the cytoplasm. These findings reinforce the assumption that deregulated Wnt/β-catenin signaling plays an important role in the promotion of EMT and local *infiltrative* cancer *growth*. In contrast, weak (≤5%) nuclear β-catenin expression was only present in 6.5% of ccRCC cases, but not in normal tissue and not associated with any clinicopathology.

## 3. Experimental Section

### 3.1. Patients and Tissue Microarrays

A total of 278 ccRCCs and corresponding normal paraffin tissue specimens from patients who underwent nephrectomy or nephron-sparing surgery at the University of Tuebingen, Germany, between May 1993 and December 2006, were included in the analysis. Informed consent was obtained according to procedures approved by the institutional review board of the University of Tuebingen (396/2012BO1). Clinical and pathological data were collected by the treating physicians and by the tumor registry database of the University of Tuebingen. Samples from pathologically representative tumor regions excluding tumor regions consisting of necrosis, fibrosis or larger vessels and adjacent benign renal tissues were used for the construction of the Tissue Microarray (TMA). Specimens were classified according to the 7th edition of the UICC/AJCC system (2009) and to Fuhrman’s grading. All samples were independently evaluated by a second histological examination of hematoxylin- and eosin-stained slides. The TMA slides were prepared with a core size of 1.0 mm; all patient probes were assembled as triplets (benign tissue 1 core, ccRCC tissue 2 cores), as previously described [40].

### 3.2. Immunohistochemical Staining and Data Analysis

Staining was performed according to the following protocol. Three micrometer sections were transferred to slides (Superfrost-Plus, Langenbrinck, Teningen, Germany). Tissues were deparaffinized by passing the specimens through xylene and rehydrated through serial dilutions of ethanol (100%, 96% and 70%). The slides were further processed in the Benchmark XT automated slide stainer (Ventana Medical Systems, Tucson, AZ, USA) using the standard antigen TBE-buffer-based antigen retrieval solution (CC1, Ventana). The β-catenin antibody (Clone CAT-5H10, Cat. No. 503-2264, Zytomed Systems, Berlin, Germany) was used at a dilution of 1/400 using antibody diluent (Cat. No. ZUC025-500, Zytomed Systems). The Wnt1 antibody (Cat. No. AB15251, Abcam, Cambridge, MA, USA) was used at a dilution of 1/100 using antibody diluent. Both antibodies were evaluated in preceding experiments to assure antibody specificity. In line with previous reports that used Western blot analysis and the recombinant proteins, both antibodies demonstrated high specific protein detection in breast carcinoma used as positive control, as well as no immunoreactivity in negative controls, with the primary antibody omitted [41]. As a detection system, the iView DAB-Detection-Kit (Cat. No. 760-091, Roche, Mannheim, Germany) was used for the β-catenin antibody and the Ultra View DAB Detection-Kit (Cat. No. 760-500, Roche, Mannheim, Germany) for the Wnt1 antibody.

Counterstaining was performed using hematoxylin. In order to exclude unspecific staining due to biotin-cross reactivity in the tumor samples, control experiments were done using a biotin blocking solution. Staining was classified according to a semi-quantitative IHC reference scale: the relative amount (0%–100%) of cells stained together with the staining intensity (0–3+) resulted in a score from 0 to 300. Results were determined as a percentage of the maximum value. Only nuclear β-catenin expression was assessed dichotomously (present *vs.* absent). TMA slides were evaluated in a blinded manner by two independent investigators. In the case of discordant scoring results, a consensus score was assigned.

### 3.3. Statistics

Continuous normally distributed variables are given as the mean value ± standard deviation, continuous non-normally distributed variables as median values with interquartile ranges and categorical data as absolute or relative frequencies. Cancer staging and grading were dichotomized into adverse (UICC/AJCC stage >pT2 and Fuhrman classification >G2) and more favorable characteristics. Wnt1, membranous, cytoplasmic and nuclear β-catenin expression scores were compared between corresponding adjacent normal and ccRCC tissue using a non-parametric test (Wilcoxon). For the analysis of clinical and pathological features and the survival analysis data were categorized into subgroups of expression: low (≤ mean) *vs.* high (> mean), according to the localization-specific mean expression scores of Wnt1 and of β-catenin, shown in Table 1. Both groups were correlated for clinical-pathological parameters using the two-sided Fischer’s exact test. Patients that were alive were censored at the time of their last follow-up. For the analysis of cancer-specific survival, all patients who did not die of cancer-specific causes were censored at the time of death. Survival curves were estimated according to the Kaplan-Meier method, and differences in survival were evaluated by the log-rank test. To identify any prognostic value, univariable Cox proportional hazard regression models were used to determine the relative risk of overall (OS) and cancer-specific survival (CSS) for each variable. In a multivariable model, including the strong predictive variables, T, N and M, and grading the independent value of Wnt1, cytoplasmic and membranous β-catenin was evaluated. A two-sided probability value <0.05 was considered to show a significant effect, although this cannot be interpreted as confirmatory. All data were analyzed with the Statistical Package for Social Sciences Software, version 18.0 (SPSS Inc., Chicago, IL, USA).

## 4. Conclusions

This study examined for the first time the tumorigenic and prognostic significance of altered Wnt1/β-catenin protein expression in ccRCC patients. Although the link between higher protein expression of Wnt1, membranous and cytoplasmic β-catenin in ccRCC samples underlines the tumorigenic importance, in this study, no corresponding increase in protein levels were detected in comparison to adjacent normal kidney parenchyma in line with all the findings on mRNA-levels [7,9].

Furthermore, in univariate analysis, only high cytoplasmic β-catenin protein expression was associated with a significantly reduced survival and lost its prognostic significance after multivariable adjustment, while the traditional histopathological parameters did not. These results obtained in patients samples are partly in conflict with cell-line based molecular experiments of the Wnt/β-catenin signal network. Although here, the sequence of high cytoplasmic β-catenin with subsequent translocation into the nucleus and activation of oncogenes seems reasonable, the mechanism of nuclear entry and the dynamic shuttling between the cytoplasm and nucleus is not precisely understood. Furthermore, the dual role of the Wnt/β-catenin as regulator of cellular homeostasis, but also adhesion in renal cancer, remains to be clarified [11].

While the value of Wnt/β-catenin in ccRCC is still controversial, Wnt antagonists already emerged as candidates for future targeted strategies. Recently, several non-specific Wnt inhibitors, including NSAIDs, retinoids, flavonoids or valproic acid, have been identified, while future therapeutic approaches aim to identify a more selective pathway inhibition by anti-Wnt antibodies or small interfering RNAs [23]. These innovative therapeutic approaches aim at a restoring the compartment depend balance of β-catenin. However, the reported results underline the complex distribution between normal and cancerous renal tissue, not to mention the problems of the localization-dependent Wnt/β-catenin blockade and the risk of harming cell homeostasis. Therefore, further studies are warranted to provide a deeper understanding of Wnt/β-catenin regulation in ccRCC patients as a basis for future targeted therapies.

## Figures and Tables

**Table 1:** Associations between Wnt1, membranous and cytoplasmic β-Catenin protein overexpression defined as immunohistochemical expression scores above the mean in clear cell renal cell carcinoma (ccRCC) tissue with tumor and patient characteristics.

Parameter	Overall (*n* = 278)	Wnt1 low # (*n* = 165)	Wnt1 high # (*n* = 106)	*p*-Value	Membranous β-Catenin low # (*n* = 186)	Membranous β-Catenin high # (*n* = 70)	*p*-Value	Cytoplasmic β-Catenin low # (*n* = 225)	Cytoplasmic β-Catenin high # (*n* = 31)	*p*-value
Age (mean ± SD; years)	62.2 (±12.5)	61.4 (±12.9)	63.9 (±11.6)	0.12	61.8 (±12.6)	64.2 (±12.3)	0.17	62.2 (±12.7)	64.1 (±11.7)	0.57
Gender (Male/Female; %)	194/84 (69.8/30.2)	108/57 (65.5/34.5)	80/26 (75.5/24.5)	0.08	134/52 (72.0/28.0)	43/27 (61.4/38.6)	0.11	157/68 (69.8/30.2)	20/11 (64.5/35.5)	0.56
Total deaths (%)	113 (41.4)	66 (59.5)	45 (40.5)	0.80	72 (67.9)	34 (32.1)	0.20	89 (84.0)	17 (16.0)	0.11
Cancer related deaths (%)	65 (23.8)	41 (64.1)	23 (35.9)	0.56	39 (66.1)	20 (33.9)	0.24	47 (79.7)	12 (20.3)	0.04 *
Tumor Diameter (mean ± SD; cm)	5.26 (±2.91)	4.9 (±2.6)	5.8 (±3.0)	0.01 *	5.3 (±2.8)	5.4 (±2.8)	0.87	5.2 (±2.7)	6.4 (±2.9)	0.01 *
Stage (T1/2 *vs*. T3/4, %)	169/109 (60.8/39.2)	114/51 (69.1/30.9)	50/56 (47.2/52.8)	0.004 *	119/67 (64.0/36.0)	34/36 (48.6/61.4)	0.03 *	144/81 (64.0/36.0)	9/22 (29.0/71.0)	0.003 *
Lymph Nodes (N0 *vs.* N1/2, %)	265/13 (95.3/4.7)	157/8 (95.2/4.8)	101/5 (95.3/4.7)	1.0	180/6 (96.8/3.2)	64/6 (91.4/8.6)	0.10	217/8 (96.4/3.6)	27/4 (87.1/12.9)	0.04 *
Distant Metastasis (M0 *vs.* M1, %)	239/39 (86.0/14.0)	140/25 (84.85/15.15)	93/13 (86.0/14.0)	0.59	159/27 (85.5/14.5)	61/9 (87.1/12.9)	0.84	193/32 (85.8/14.2)	27/4 (87.1/12.9)	1.0
Grade (G1/2 *vs.* G3/4, %)	234/44 (84.2/15.8)	136/29 (82.0/17.6)	92/14 (86.8/13.2)	0.40	162/24 (87.1/12.9)	53/17 (75.7/24.3)	0.04 *	199/26 (88.4/11.6)	16/15 (51.6/48.4)	≤0.001 *
Vascular invasion (no/yes, %)	193/85 (69.4/30.6)	124/41 (75.15/24.85)	64/42 (60.4/39.6)	0.02 *	137/49 (73.7/26.3)	39/31 (55.7/44.3)	0.01 *	163/62 (72.4/27.6)	13/18 (41.9/58.1)	0.002 *
Perinephric Invasion (%)	47 (16.9)	157/8 (95.1/4.9)	94/12 (88.7/11.3)	0.06	171/15 (91.9/8.1)	66/4 (94.3/5.7)	0.60	208/17 (92.4/7.6)	29/2 (93.55/6.45)	1.0
Sinus Invasion (%)	65 (23.4)	143/22 (86.7/13.3)	89/17 (84.0/16.0)	0.60	157/29 (84.4/15.6)	62/8 (88.6/11.4)	0.55	194/31 (86.2/13.8)	25/6 (80.65/19.35)	0.42
Necrosis (%)	114 (41.0)	100/65 (60.6/39.4)	59/47 (55.7/44.3)	0.45	114/72 (61.3/38.7)	33/37 (47.1/52.9)	0.04 *	133/92 (59.1/40.9)	14/17 (45.2/54.8)	0.18
Sarcomatoid features (%)	19 (6.8)	152/13 (92.1/7.9)	100/6 (93.0/7.0)	0.63	175/11 (94.1/5.9)	63/7 (93.0/7.0)	0.28	213/12 (94.7/5.3)	25/6 (80.65/19.35)	0.01 *

# the immunohistochemical staining score in tumor samples was used as a cut-off to define low (≤mean) and high (>mean) protein expression;

* indicates significance *p* < 0.05.

**Table 2:** Univariate analysis by Cox proportional hazards model of clinicopathological parameters on cancer-specific and overall survival in 278 ccRCC patients.

		Hazard Ratio (95% CI)			
Variable	Categories	Overall survival	*p*-value	Cancer specific survival	*p*-value
Age	±median	1.69 (1.15–2.48)	0.007	1.00 (0.62–1.64)	0.97
Gender	female *vs.* male	0.90 (0.59–1.26)	0.6	0.58 (0.31–1.07)	0.08
T-stage	T1/2 *vs.* T3/4	4.32 (2.93–6.37)	<0.0001	9.62 (5.20–17.77)	<0.0001
N-stage	N0 *vs.* N1/2	3.22 (1.68–6.19)	<0.0001	5.01 (2.50–10.20)	<0.0001
M-stage	M0 *vs.* M1	5.51 (3.59–8.47)	<0.0001	9.57 (5.65–16.19)	<0.0001
Nuclear Grade	G1/2 *vs.* G3/4	3.80 (2.52–5.72)	<0.0001	6.78 (4.13–11.13)	<0.0001
Tumor Necrosis	yes *vs.* no	1.80 (1.24–2.60)	0.002	2.81 (1.70–4.66)	<0.0001
Sarcomatoid Differentiation	yes *vs.* no	4.60 (2.72–7.76)	<0.0001	7.36 (4.08–13.25)	<0.0001
Vascular Invasion	yes *vs.* no	3.10 (2.13–4.50)	<0.0001	5.67 (3.39–9.50)	<0.0001
Perinephric Invasion	yes *vs*. no	2.53(1.23–4.11)	0.008	2.20 (1.00–4.84)	0.5
Sinus Invasion	yes *vs.* no	2.29 (1.46–3.58)	<0.0001	2.78 (1.59–4.85)	<0.0001
Wnt1 Score tumor	±mean	1.18 (0.81–1.73)	0.4	0.95 (0.57–1.58)	0.84
β-catenin Score membrane tumor	±mean	1.26 (0.84–1.89)	0.27	1.35 (0.79–2.30)	0.28
β-catenin Score cytoplasm tumor	±mean	1.75 (1.04–2.94)	0.035	2.26 (1.20–4.27)	0.012

**Table 3:** Multivariate Cox proportion hazard model for cancer-specific and overall survival in ccRCC patients.

		Hazard Ratio (95% CI)			
		
Variable	Categories	Overall survival	*p*-value	Cancer specific survival	*p*-value
Age	**±**median	1.88 (1.25–2.83)	0.002	1.18 (0.69–2.00)	0.56
T-stage	T1/2 *vs.* T3/4	2.63 (1.63–4.23)	<0.0001	4.30 (2.03–9.10)	<0.0001
N-stage	N0 *vs.* N1/2	1.59 (0.75–3.42)	0.23	1.89 (0.83–4.32)	0.13
M-stage	M0 *vs.* M1	3.43 (2.09–5.62)	<0.0001	4.58 (2.46–8.52)	<0.0001
Nuclear Grade	G1/2 *vs.* G3/4	0.98 (0.54–1.80)	0.96	1.37 (0.69–2.73)	0.37
Tumor Necrosis	yes *vs.* no	1.23 (0.83–1.84)	0.3	1.65 (0.94–2.90)	0.08
Sarcomatoid Differentiation	yes *vs.* no	1.63 (0.83–3.20)	0.15	1.62 (1.68–3.45)	0.16
β-catenin Score cytoplasm tumor	±mean	1.00 (0.55–1.84)	0.99	0.88 (0.43–1.83)	0.75
